# Heat Treatment of Milk: A Rapid Review of the Impacts on Postprandial Protein and Lipid Kinetics in Human Adults

**DOI:** 10.3389/fnut.2021.643350

**Published:** 2021-04-30

**Authors:** Mona Fatih, Matthew P. G. Barnett, Nicola A. Gillies, Amber M. Milan

**Affiliations:** ^1^Polytech School of Engineering, University of Angers, Angers, France; ^2^AgResearch Ltd., Grasslands Research Centre, Palmerston North, New Zealand; ^3^Riddet Institute, Hosted by Massey University, Palmerston North, New Zealand; ^4^The Liggins Institute, The University of Auckland, Auckland, New Zealand

**Keywords:** lipid, protein, postprandial, dairy, pasteurized, ultra-high temperature

## Abstract

**Background:** Most milk consumed by humans undergoes heat treatment to ensure microbiological safety and extend shelf life. Although heat treatment impacts the structure and physiochemical properties of milk, effects on nutrient absorption in humans are unclear. Therefore, a rapid review was performed to identify studies conducted on healthy human adult subjects that have assessed the impacts of heat treatment of milk on protein and fat digestion and metabolism in the postprandial period (up to 24 h).

**Methods:** Relevant databases (Medline, EMBASE, Cochrane, Scopus) were systematically screened for intervention studies on healthy adult men and women that assessed the impact of consuming heat-treated milk on the postprandial kinetics or appearance in peripheral circulation or urine of ingested proteins and/or lipids. The risk-of-bias assessment tool 2 was used for quality assessment.

**Results:** Of 511 unique database records, 4 studies were included encompassing 6 study treatments (*n* = 57 participants, 20–68 years). Three studies evaluated pasteurization, two evaluated ultra-high temperature (UHT) treatment, and one evaluated oven-heated milk. Protein and lipid appearances in peripheral blood were reported in two sets of two studies. None of the studies used the same heat treatments and outcome measures, limiting generalization of effects. Protein appearance (ng/mL or area under the curve) (as plasma amino acids - lysine) was reduced when milk was oven-heated for 5 h in one study (*n* = 7 participants), while the other study reported a reduced retention of dietary N with UHT milk (*n* = 25 participants). Overall plasma triacylglycerol responses were unaffected by milk heat treatments reported, but plasma fatty acid composition differed. The studies observed higher plasma myristic and palmitic acid abundance with successive heat treatment at 2 h (*n* = 11 participants; pasteurized) and 4 h (*n* = 14 participants; UHT) after ingestion; other differences were inconsistent. All studies had moderate-high risk of bias, which should be taken into consideration when interpreting findings.

**Discussion:** This review identified few studies reporting the effects of milk heat treatment on postprandial nutrient responses in adults. Although the findings suggest that milk heat treatment likely affects postprandial protein and lipid dynamics, generalization of the findings is limited as treatments, outcomes, and methods differed across studies. Because of the study variability, and the acute post-prandial nature of the studies, it is also difficult to draw conclusions regarding potential long-term health outcomes. However, the possibility that altered digestive kinetics may influence postprandial protein retention and anabolic use of dietary N suggests heat treatment of milk may impact outcomes such as long-term maintenance of muscle mass.

## Introduction

Heat treatment is a widely used technique in the dairy industry, as fresh liquid milk is a product with a short shelf-life. In addition, although the careful use of animal husbandry and on-farm hygiene practices enables raw milk of high microbiological quality to be obtained, raw milk can be microbiologically unsafe for human consumption (1, 2), due to the presence of harmful bacteria including *Campylobacter, Salmonella*, Shiga toxin-producing *Escherichia coli* (3) and *Listeria monocytogenes* (1, 2). Heat treatment has been defined as follows by the International Dairy Federation: “Any intentional heating above 50 °C for a sufficient time such that there is a reduction in the concentration of one or more microorganisms is considered heat treatment” (4). Standard heat treatment includes high-temperature short time pasteurization (72 to 80 °C for 15 to 30 s) and ultra-high temperature (UHT) processing (135 to 150 °C for 1 to 10 s) (4), with the primary objective to make milk safe for human consumption.

With a view to understanding the impact of milk processing on human health, investigations of the consequences of processing on milk structure and physical properties have been conducted using *in vitro* systems (5–7). Pasteurization and UHT processing were shown to increase the aggregation of fat and protein into a semisolid curd under simulated gastric conditions (5). UHT milk was shown to have faster rates of protein hydrolysis compared to pasteurized milk and a faster release of fat globules during digestion. Those results were explained by differences in the structure of the different milk curds (6). Protein modifications occurring during heating processes (i.e., pasteurization) have been shown to impact protein composition and function of the milk fat globule membrane, which have the potential to impact digestion (8). Those structural changes, especially in UHT milk, include a looser curd structure, creating a larger surface area for the diffusion of pepsin into the curd (7). A smaller weight of milk curd can lead to a quicker protein hydrolysis and thus, speed up the gastric emptying process (6). Moreover, a rodent study assessing the impact of different thermal treatments (UHT, pasteurized, and spray-dried milks) on the bioavailability of dairy proteins showed a modification of the postprandial splanchnic protein extraction and a small but significant impairment in digestibility following spray-dried milk, with no impact of heat treatment on nitrogen availability. The authors concluded that altered postprandial metabolism may relate to the degree of protein lactosylation (9); lactosylation is enhanced by heat treatment (10).

However, the impact of heat treatment on the ways in which proteins and lipids are absorbed in humans have not been detailed by these *in vitro* or animal studies. Our objective was to identify studies conducted on healthy human adult subjects that have assessed the impacts of heat treatment of milk on protein and fat digestion in the postprandial period (up to 24 h), using non heat-treated milk as a comparison across studies where possible. For this purpose, we used a rapid review methodology to review the literature.

## Methods

A rapid review methodology was used to complete this study. The objective of a rapid review is to synthesize knowledge in order to produce information under time constraints by simplifying the systematic review process. Systematic reviews usually take many months, or even up to years to produce, whereas rapid reviews typically only take from 1–6 months, and may limit the scope of sources searched (e.g., gray literature) (11, 12).

Using the Population Intervention Comparator Outcome Time (PICOT) format (13), our research question was formulated as follows: Are there differences in protein and/or lipid digestion and metabolism in the 24 h following consumption of different types of heat-treated milk, or heat-treated compared to non-heat-treated milk, among adult men and women (18–70 years)? (Table 1).

**Table 1:** PICOT criteria employed to define the research question.

**Criteria**	**Description**
Participants	Human men and women (18–70 years)
Intervention	Heat-treated milk
Comparison group	Non-heat-treated milk, or milk undergoing a different heat treatment
Outcome of interest	Parameters of protein and/or lipid digestion or metabolism
Time	≤ 24 h following ingestion of milk

### Inclusion Criteria

The inclusion criteria (Table 2) were determined by one reviewer (MF) and then shared for approval with the second reviewer (AM).

**Table 2:** Inclusion and exclusion criteria applied in article screening.

	**Inclusion**	**Exclusion**
Population	Healthy human participants Men and women Adults (18–70 years)	Animals Population with chronic diseases (e.g., cardiovascular diseases, cancer) metabolism disorders (e.g., thyroid) or gastrointestinal diseases (e.g., celiac disease, inflammatory bowel disease) Populations with known intolerance to milk
Study design	Interventional Follow up maximum 24 h	Observational Study cases Interventions over 24 h long
Study setting	Clinical trial	Community
Intervention	Consumption of a meal with milk that was heat treated (e.g. UHT, pasteurization, ESL)^1^	If milk was not heat treated and only went through another type of process (e.g., microfiltration, homogenization)
Outcome	protein and/or lipid kinetics; protein and/or lipid appearance and clearance in blood circulation and/or urine	
Publication status		Full text is not available in English

*According to the International Dairy Federation, “Any intentional heating above 50° C for a sufficient time such that there is a reduction in the concentration of one or more microorganisms is considered heat treatment” (4). ESL, extended shelf life; UHT, ultra-high temperature*.

### Search Strategy

The electronic databases Medline (ovid), EMBASE (ovid/ 1980-present), The Cochrane Library and Scopus were searched without any restrictions to the time of publication. The search was carried out from June 10–16th 2020. The research question was separated in four concepts grouping all the Medical Subject Headings (MeSH) and keywords relevant to each concept. All those terms were linked within each concept with the connector OR and the four concepts were joined using the connector AND. The MeSH terms were adapted according to the specificity of each database. No MeSH terms were used to search Scopus but only the keywords in the title, abstract and keywords of the database. The full search strategy and its adaptation for each database can be found in Supplemental Tables 1–4.

All the search results were exported to the reference manager Mendeley (v1.19.4 Elsevier Inc., Amsterdam, Netherlands) where duplicate articles were removed.

### Screening Process

Titles and abstracts of articles were screened independently by two reviewers (MF and AM) against the inclusion and exclusion criteria. The discrepancies were resolved through discussion, or by consultation with a third reviewer if necessary (NG). If the decision to include an article was unclear at any stage, then the full text was obtained. Next, the full text of relevant articles was obtained and screened to ensure their eligibility.

### Data Extraction

The two reviewers independently extracted the following information from the relevant full-text articles: author, date and country of the article, purpose of the study, type of heat treatment, temperature and length of the treatment, milk of comparison, volume of milk, time of follow-up, number, sex and age range of the participants, outcome measurement and results.

### Quality Assessment

The risk of bias was assessed using the Cochrane risk-of-bias assessment tool for randomized trials (RoB 2) (14). The risk of bias was assessed (by MF) for each independent outcome within a study where differences between the outcomes were observed (e.g., missing data for a specific outcome, different number of analyzed samples).

## Results

### Study Selection

**Figure 1:**
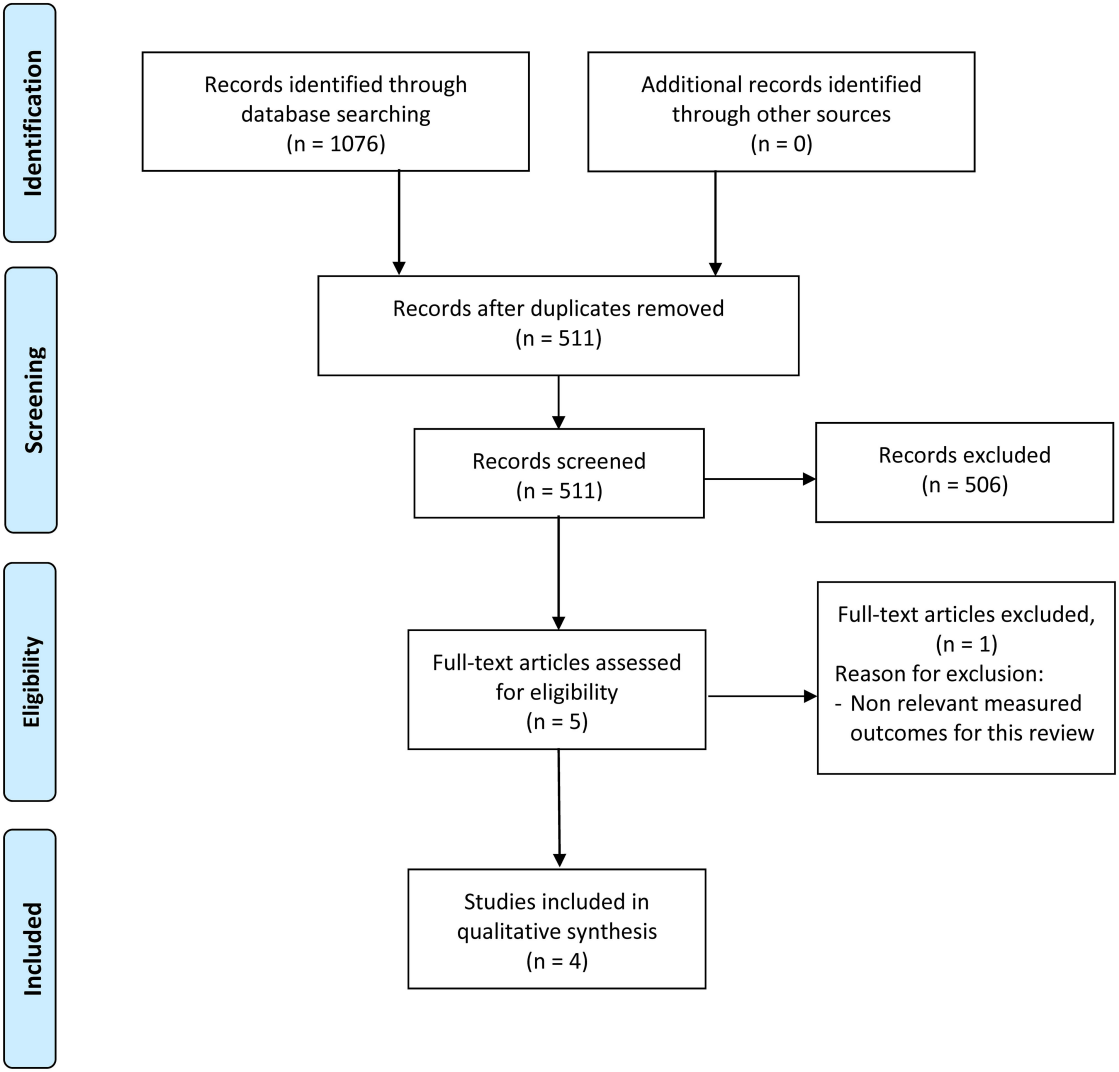
PRISMA flow diagram outlining search results.

A total of 1,076 articles were identified from the four database searches and exported to Mendeley. The duplicate check was then completed, resulting in 511 articles. After the title and abstract screening, 506 articles did not match the inclusion criteria and were excluded from full-text review. Finally, we assessed the full-text of 5 articles, from which 1 was excluded because the measured outcomes did not meet the inclusion criteria (Figure 1).

### Study Characteristics

Two of the studies were conducted in Finland by the same group (15, 16). One was conducted in France (17), and one in Sweden (18). One study was a randomized parallel trial (17) and the other three studies were randomized cross-over trials (15, 16, 18). In total, *n* = 57 subjects were included. All studies were conducted on both male and female subjects. Most subjects were aged from 20 to 40 years old but one study reported their eldest subject to be 68 (15). All the subjects were reportedly healthy. In two of the studies (15, 16), subjects reported gastrointestinal (GI) symptoms after drinking milk but no lactose intolerance had been previously diagnosed. Across the four studies, there were six treatment groups in which heat-treated milks were consumed. Three studies measured the effects of pasteurization (15–17), two measured the effects of UHT treatment (16, 17) and in one study the milk was heated in an oven (18). Measured outcomes were glycemia in three of the studies (15–17), insulinemia, lipemia, inflammation and GI symptoms in two of the studies (15, 16), amino acids (AAs) in plasma in one study (18) and AAs, protein and urea in serum, urinary creatinine, urinary urea and dietary N incorporation in one study (17) (Table 3).

**Table 3:** Characteristics of included studies assessing postprandial protein or lipid responses to heat treated milk in adults.

		**Lacroix et al. (2018) (17)**	**Ljungqvist et al. (1979) (18)**	**Nuora et al. (2018a) (15)**	**Nuora et al. (2018b) (16)**
Study characteristics	Country	France	Sweden	Finland	Finland
	Study objective	To assess impact of heat treatment (microfiltration, Pasteurization, UHT) on protein quality (measured by nitrogen metabolism) following single meal	To assess impact of lactose hydrolysis of skim milk powder on lysine availability (in heat treated samples)	To assess impact of native milk v homogenized & pasteurized milk on gastrointestinal symptoms, inflammation, transit, intestinal pressure, glycemia, insulinemia or lipemia	To assess impact of milk processing on gastrointestinal symptoms, inflammation, lipemia, glycemia, insulinemia
Participant characteristics	*n*	25 (11M; 14F)	7 (3M; 4F)	11 (5M; 6F)	14 (6M; 8F)
	% male	44%	43%	45%	43%
	Age range (years)	Age range not reported, range of means: 23.5 ± 6.9–27.1 ± 7.8	24–29	24–68	20–45
Heat treatment	Type	1. Pasteurized 2. UHT	Lactose hydrolyzed freeze-dried skim milk heated in oven	Homogenized pasteurized	1. Homogenized pasteurized. 2. Homogenized UHT
	Temperature	Pasteurization: 72 °C; UHT: 140°C	66 °C	72–73 °C	Homogenized Pasteurization: 73 °C; Homogenized UHT: 135 °C
	Length of treatment	Pasteurization: 20 s; UHT: 5 s	1, 3, and 5h	15 s	Homogenized Pasteurization: 15 s; UHT: 3 s
Comparison milk		Microfiltered	Lactose hydrolyzed freeze-dried skim milk without oven-heat treatment	Raw milk	Pasteurized^*a*^
Intervention	Study protocol prior to testing	Standardized diet, adjusted to participant's body weight, was provided for one-week prior to the study day. Overnight fast prior to testing.	Overnight fast prior to testing.	Non-dairy diet for 5 days prior to the study day. Overnight fast prior to testing.	Non-dairy diet for 5 days prior to the study day. Overnight fast prior to testing.
	Test meal	No test meal consumed with the milk.	Milk samples were mixed with gluten (1:1 on basis of protein content)	SmartBar (Given Imagine, Israel)	Rice cakes (24g), turkey cold cuts (85g), cucumber (50g)
	Volume of milk consumed	500 mL	400 mL	400 mL	400 mL
	Duration of follow-up	8 h	2 h	4 h	5 h
	Frequency of sampling	30, 60, 90, 120, 150, 180, 240, 300, 360, 420, and 480 min after ingestion.	2h after ingestion.	20, 40, 60, 90, 120, 180, and 240 min after ingestion.	20, 40, 60, 90, 120, 180, 240, and 300 min after ingestion.
Milk characteristics	Fat content - test	Defatted milk	Not reported	Not reported	Not reported
	Fat content - control	Defatted milk	Not reported	Not reported	Not reported
	Protein content - test	23.3g	Not reported	34.6 ± 0.6 g/kg	Not reported
	Protein content - control	23.3g	Not reported	34.8 ± 1.7 g/kg	Not reported

a *Pasteurized milk was heated under the same conditions as the homogenized pasteurized milk, but did not undergo earlier homogenization at 16 MPa before heat treatment. F, female; UHT, ultra-high temperature*.

### Risk of Bias Within Studies

Three of the studies were assessed with some concerns on the overall bias (15–17) and one was assessed with a high risk of bias (18).

**Figure 2:**
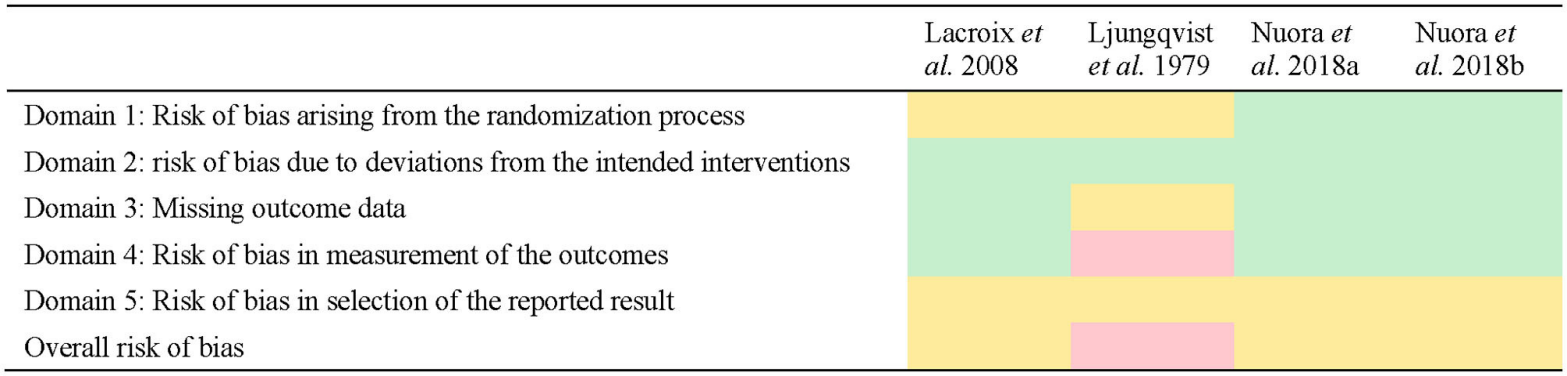
Quality assessment of studies using the Risk of Bias 2 tool. Green indicates low risk, yellow indicates some concerns, red indicates high risk.

These findings reflected a lack of reporting within the methods used in the study by the investigator team (e.g., lack of reporting of a randomization strategy or a pre-specified analysis plan before having the results). For instance, pre-specified analysis plans were not specified or available for all studies, meaning that none of the included studies could satisfy a low risk of bias under Domain 5 of the tool. No differences in risk assessment were found within studies reporting multiple outcomes, so risk of bias has been presented only once per study (Figure 2).

### Summary of Study Findings

#### Proteins and Amino Acids

The study that measured the plasma AA response 2 h after ingestion of milk heated in the oven [Ljungqvist et al. (18)] found a significant drop in the lysine plasma concentration between the milks heated for 0 h (−3.9%) and 5 h (−10.1%); hence, compared to raw milk (0 h) the lysine plasma concentration was lower. However, the study by Lacroix et al. that assessed the plasma AA concentrations after the ingestion of micro filtered milk (MF), pasteurized milk (PM) and UHT-treated milk (17) did not report any significant differences across plasma AA, including lysine plasma concentration. Lacroix et al. also reported significant differences between the time courses of dietary N transfer into serum AA, urea N, and protein pools, using ^15^N milk labeling. UHT showed 5% higher dietary N in the body urea pool after 8 h than MF and PM; in serum proteins this elevation was ~1% higher. A significant meal effect was observed after the consumption of UHT milk compared to PM and MF milk (Table 4). A postprandial reduction in dietary N retention of 8% was also observed after ingestion of the UHT compared to the two other types of milk (Table 4).

**Table 4:** Findings of included studies assessing postprandial protein or lipid responses to heat treated milk in adults.

	**Lacroix et al. 2008**	**Ljungqvist et al. 1979**	**Nuora et al. 2018a**	**Nuora et al. 2018b**
Outcome measurements	Blood glucose serum urea, AA, protein, N urinary creatinine & urea, ammonia, N N incorporation into body pools	Plasma AA% molar ratio of AA	Gastrointestinal symptoms, inflammation, transit, intestinal pressure, glycemia, insulinemia, lipemia/fatty acids	Gastrointestinal symptoms, inflammation, lipemia, glycemia, insulinemia
Significant results	Dietary N: body urea: 15% UHT v 10% MF/PM serum proteins: 7.7 ± 1.2% UHT v 6.1 ± 1.0% MF v 6.4 ± 1.5% PM urinary urea: 11.7 ± 3.1% UHT v 8.0 ± 2.1% MF v 8.1 ± 2.4% PM 8 h in body urea: 25.9 ± 3.3% UHT v 18.5 ± 3.0% MF v 18.6 ± 3.7% PM NPPU: MF = PM, UHT lower by 8% UHT greater N loss	Limited plasma lysine & sulfur AA in all heat-treated milk v unheated: lysine plasma AA at −3.9 at 0 h and at −10.1 at 5 h	Higher plasma myristic, palmitic, stearic acid 4 h after HPM than NM	Higher plasma myristic, palmitic, oleic, linoleic 2 h after UHT or PM than HPM linoleic higher PM than UHT, also UHT higher than HPM at 4 h
Quality assessment	Some concerns	High risk	Some concerns	Some concerns

*AA, amino acids; F, female HPM, homogenized pasteurized milk; MF, microfiltered milk; M, male; N, nitrogen; NM, native milk; NPPU, net postprandial protein utilization PM, pasteurized milk; UHT, ultra-high temperature*.

#### Lipids

No significant differences were found in the plasma triacylglycerol concentrations between homogenized, pasteurized and UHT milk (16), nor in the blood triacylglycerol concentrations between raw milk (“native milk”; NM) and homogenized and pasteurized milk (HPM) (15). However, significant differences were found in the fatty acid composition of plasma lipids. Myristic, palmitic, and stearic acids were found to be higher 4 h after the consumption of the HPM compared to the NM. However, no significant differences were observed at the 2 h time point (15). In the other study measuring this outcome (16), significant differences were also reported. At the 2 h time point, myristic, palmitic, oleic and linoleic acids were found to be significatively higher after the UHT milk and the PM compared to the HPM. At the 4 h time point, linoleic acids levels were found to be higher after PM compared to the UHT milk that was itself higher than the HPM.

#### Plasma Glucose and Insulinemia

The three papers that measured plasma glucose concentration after drinking the milks did not report any significant differences between milks processed by different methods regarding that outcome (15–17). No significant differences were reported for plasma insulin levels either (15, 16).

## Discussion

The purpose of this review was to identify studies that assessed the impact of heat treatment of liquid milk on protein and lipid digestion and metabolism in human adults. A total of four papers reporting studies on healthy human subjects were included, of the 511 initially identified. All studies were assessed as having moderate-high risk of bias. Two of the studies reported outcomes related to lipids (15, 16) and the other two reported outcomes related to proteins (17, 18). Despite the topic of milk heat treatment being relevant for commercial milk products (3) and consumer nutritional preferences (19, 20), we did not find many studies measuring our outcomes of interest (i.e., protein and fat metabolism after consumption) directly in human subjects.

Our search resulted in 511 articles to screen, although only four articles met the inclusion criteria, all of which described studies that were conducted in Europe. Most were excluded for reasons such as being conducted on animals (9, 21, 22) or infants (23–25), only assessing physical and technological milk properties (26) (e.g., rheological and thermal properties, microstructure), participants drinking milk protein mix (e.g., whey beverages) but not plain milk (27, 28), studies based on plant-based milk (29, 30), or measured outcomes which were not relevant for this review (e.g., vitamins, iron, zinc) (31). Indeed, a recent systematic review of the processing impacts on milk protein digestion (32) identified only two human studies meeting their criteria, of which only one [Lacroix et al. (17)] investigated liquid milk (in this case, defatted) and is therefore common to our comparison. The large number of irrelevant articles may have been avoided with refinement of search terms; however, the current search strategy also highlighted areas which may be suitable for future reviews into the effects of heat treatment of milk on digestion in contexts other than liquid milk or human populations. As only four studies were included in the final assessment, this suggests either a lack of studies conducted specifically on adult human subjects for the specified outcomes or a lack of additional search terms that could be relevant. For instance, terms such as *in vivo*, nitrogen metabolism, or alternate terminology for heat treatments may have been missed, in addition to possible content available in the gray literature.

Half of the studies were conducted on self-described milk intolerant people but with no clinically diagnosed intolerance (15, 16). GI symptoms were indeed described by study participants. It is unclear whether the inclusion of participants who experience symptoms such as bloating or cramping may have influenced the outcome measures of digestion, and it is not possible to compare and contrast evidence due to the small number of studies conducted. We did not take into consideration the reported outcomes of those studies regarding GI symptoms and inflammation markers as they were not outcomes of interest for this review. Although there is some evidence that plasma AA appearance after milk ingestion is unimpaired in subjects with lactose intolerance, other forms of dairy intolerance may impact plasma AA (33) or other micronutrient responses (34). Conducting studies on subjects that do not experience GI symptoms would be the next step in getting a better understanding of how heat processing influences the composition of circulating fatty acids, as both studies evaluated in this review included subjects with reported GI symptoms.

Only one study assessed outcomes related to protein metabolism (17) with this study also investigating serum AA dynamics. This showed that protein digestion may be accelerated with heat treatment, in this case UHT, with this change in digestive dynamics potentially driving enhanced anabolic use of dietary N specifically in serum proteins. However, this study also showed that net postprandial protein utilization (NPPU) was significantly reduced in the UHT group, indicating that more nitrogen/amino acids were oxidized in the UHT treatment. This suggests that overall protein retention was lower, and on a whole-body level the lower NPPU of the UHT treatment group reflects a reduced anabolic effect. One other study also assessed AA dynamics in plasma (18). In this case, AA concentrations at 2 h were lower with heat-treated milk (oven heated) *vs*. raw milk, suggesting reduced AA availability after heat treatment. However, it is also possible that the lower AA concentrations after heat treatment may reflect more rapid clearance from plasma and use for protein synthesis. The impacts of heat treatment on protein digestion have been assessed *in vitro* (6) and in animals (9, 21, 35), and indeed, many studies that were excluded from the review assessed protein digestion yet in the context of derivatives of liquid milk (36) or in non-adult populations (23, 24). Yet, few studies have measured protein or amino acid dynamics in human adult subjects. Given the large body of evidence to support protein modifications to dairy products *in vitro* and in animal models [as reviewed by van Lieshout et al. (32)], it is perhaps surprising so few human studies exist.

The quality assessment indicated that some concerns on the risk of bias existed in three of the studies (15–17) with one study having a high risk of bias (18). The study by Ljungqvist et al. (18) was considered to have a high risk of bias primarily because the number of samples included in the results did not always match the number of participants, nor were they consistent at each time point, and no explanation was provided for this discrepancy. The study design and implementation was also unclear (e.g., no information is provided about the randomization of the sequence, if the trial is blind or double-blind) and may in part be explained by the time the study was conducted (c. 1979) relative to the introduction of standard reporting guidelines such as Consolidated Standards of Reporting Trials (CONSORT) (37). Indeed, most of the studies may have had higher quality assessment scores if the research methodology had been reported more thoroughly. In many cases, domains were scored with at least some concerns on the risk of bias because information was missing (e.g., a clear explanation on how the randomization sequence was obtained, no indication of whether the outcomes were measured on all the participants, reporting of the subject characteristics). Taking the RoB 2 tool guidelines (14) into account when determining the design of a randomized trial and then when reporting the methodology would help to avoid any doubts on the reliability of the trial's results. The quality of the three most recent papers (15–17) appears to be satisfactory in their lower risk of bias to provide a reliable quality of evidence due to their detailed reporting of the milk characteristics, inclusion and exclusion criteria, experimental design and methods of analysis of the outcomes.

The overall findings suggest significant differences in the plasma fatty acid composition due to milk processing methods, including greater plasma concentrations of myristic and palmitic acids after UHT relative to HPM (16), and relative to NM (raw milk) (15). However, it is important to note that the two studies assessing this outcome (15, 16) were conducted by the same group of researchers so similarities in methodology used could contribute to the alignment of these findings. Moreover, each study made comparisons between different types of milk processing, so it is not possible to determine whether consistent results have been observed across studies for any specific milk processing type. Subjects also did not have their diet standardized, apart from not consuming dairy products 5 days before the trial. Participants of both studies also experienced GI symptoms after milk consumption such as cramping or bloating (15, 16). It is possible that these symptoms could influence the findings of nutrient appearance in blood circulation. There is evidence that GI symptoms (38, 39), as well as GI disorders including inflammatory bowel disease, Crohn's disease and celiac disease (40–42) are associated with reduced absorption of some nutrients. GI symptoms such as diarrhea or constipation are also known to be characterized by changes to GI transit times (43); however, the extent to which these symptoms impact small intestinal absorption, or whether these factors influences any of the reported findings is not clear. These studies were not able to determine the mechanisms for blood circulating fatty acid composition differences at specific time points following consumption of processed milks. It is still unclear how the lipid droplet size differences observed after *in vitro* digestion of pasteurized cream (22) influences the intestinal absorption of lipids. Therefore, conducting a study on a bigger group of people, standardizing diets prior the trial, ensuring subject homogeneity, etc. could help to limit variability and remove some of the sources of possible uncertainties that could influence the results.

Regarding measures of protein digestion and metabolism, results were not conclusive due to the limited number of studies available, and the variation in outcome measures used. Yet, both studies reported findings on both sexes, of similar ages (~20–30 years), and consuming similar quantities (400 vs. 500 mL). The study with a high risk of bias, non-commercial heating methods, and shorter follow-up (2 h) (18) reported a significant loss in the plasma lysine concentration with milk heated for a longer period of time, but those results did not match a more recent study with a better quality assessment, standard processing (i.e., PM and UHT) and longer follow-up (8 h) (17) that reported no significant changes in the serum AAs or in the lysine serum concentration with UHT milk relative to MF or PM. The latter study also provided a separated analysis of dispensable (non-essential) AAs, indispensable (essential) AAs and lysine concentration in the serum, although, no further information about the AA composition in serum was provided. Further, the heat treatment of the milks between studies differed: oven heated at 66°C for 1–5 h [lower temperature and longer duration than standard processing methods (32, 44)] vs. UHT, pasteurized and homogenized milk. These differences limit the ability to compile the effects of any one type of heat treatment on AA appearance, and in terms of relevance to commonly consumed products, data were available from only a single study.

This review highlights the current lack of studies investigating the impacts of milk heat treatment on protein and lipid digestion involving human adult participants. *In vitro* studies have been able to demonstrate that processing affects milk structure and thus digestion by changing the rates of protein hydrolysis and the release of milk fat (5–7, 32) but this is not yet the case for *in vivo* studies on humans. Variability in methodologies used further complicates the ability to draw consensus conclusions across the studies that were identified. Across studies, the milks underwent various heat treatment methods, with few comparable comparisons between the studies. One study compared UHT milk and pasteurized and homogenized milk with pasteurized milk only (16); no non-heated milk was used as a control. Three other studies used non-heated milk as a control; however, different processing techniques were still used. One study used raw milk (as NM) (15), another microfiltered milk (17) and one study used lactose hydrolyzed freeze-dried skim milk (18). Indeed, not having the same control makes it more difficult to compare findings and provide confident summaries. Likewise, the methods used to assess study outcomes, particularly protein and amino acid kinetics, were diverse. Only one study measured dietary N transfer and showed that UHT processing reduced this outcome compared to pasteurization and microfiltration (17). While this suggests heat treatment may have implications for long-term health outcomes relative to protein utilization, none of the other studies included in this review assessed this outcome so further investigation is required to clarify the impact that the different heat treatments have on the use and retention of ingested milk proteins.

This review has a number of strengths. The objective of the study was developed based on an established methodology (PICOT) and the search is well-described and rigorous, including a large amount of words and terms relevant for the objective. In addition, two of the authors did individual screening of the search results. A quality assessment was performed using the Cochrane risk-of-bias assessment tool for randomized trials, to provide an indication of the quality of research on which any conclusions were based. Finally, we have undertaken a critical discussion of the findings.

We acknowledge several limitations of the review. First and foremost, very few relevant studies were found, and the number of study subjects was relatively small, making it difficult to draw any clear conclusions. However, we believe this is in itself interesting, as it demonstrates that relatively little research has addressed the posed question, and this therefore clearly indicates that further research is required. It is also possible that content available in the gray literature may have proven useful, although given the nature of this rapid review a conscious decision was made to exclude such literature.

Because of the small number of studies identified, and the range of different heat treatment regimes used in these studies, it was also not possible to make a meaningful comparison of particular heat treatments, and we also note this as a limitation.

Although we believe this was an extensive search, some key words that could be considered relevant were not included, for example “apoB-48” and “chylomicrons” which are relevant to lipid digestion. However, we note that a subsequent search including these terms did not identify any additional studies.

## Conclusion

The present review showed an overall shortage of studies conducted on this topic on adult human subjects; as such, no solid conclusions about the impact of heat treatment of milk on protein and lipid digestion can be made. Differences between types of milk heat treatment could be shown on plasma fatty acid composition by the different articles but limitations to those findings prevented any conclusion about the way lipids and fatty acids are metabolized. No conclusive results could be obtained about how heat treatment affects postprandial protein metabolism. The lack of conclusive findings regarding both lipid and protein metabolism also makes it difficult to draw conclusions regarding long-term health impacts of consuming milks undergoing different heat treatments, although there may be implications on long-term protein utilization. Therefore, the main finding from this work is that further investigation is required to link the effects of heat treatment observed *in vitro* to *in vivo* observations, and to understand the relevance to human health.

## Data Availability Statement

The original contributions presented in the study are included in the article/Supplementary Material, further inquiries can be directed to the corresponding author.

## Author Contributions

MF designed and conducted the research, analyzed data, and wrote the paper. MPGB wrote the paper. NG conducted the research and wrote the paper. AMM designed and conducted research, analyzed data, and wrote the paper and had primary responsibility for final content. All authors contributed to the article and approved the submitted version.

## Conflict of Interest

MPGB and AMM are current employees of AgResearch Limited. The remaining authors declare that the research was conducted in the absence of any commercial or financial relationships that could be construed as a potential conflict of interest.
