# Supplementary material for: Heat Treatment of Milk: A Rapid Review of the Impacts on Postprandial Protein and Lipid Kinetics in Human Adults
Source: Front Nutr. 2021 Apr 30;8:643350. doi: 10.3389/fnut.2021.643350 (PMC8119631; doi:10.3389/fnut.2021.643350)
Supplement: Supplementary file 1 [file Table_1.DOCX]

Supplementary Material

# Supplementary Tables

**Supplemental Table S1** Medline (ovid) search strategy

|  | Concept 1 | Concept 2 | Concept 3 | Concept 4 |
| --- | --- | --- | --- | --- |
| Concept | Milk | Heat treatments | Proteins and fats absorption | In the next 24 hours |
| Mesh headings | Milk  Milk protein  Dietary proteins  Dietary fats | Hot temperature  Sterilization  Pasteurization  Food preservation | Digestion/ Gastrointestinal absorption/ Intestinal absorption/ Gastric absorption/ Absorption, physiological /Absorption/ Proteolysis/ Gastrointestinal motility/ Dietary proteins/ Amino acids/ Blood proteins/ Kinetics/ Metabolism/ Biological Availability/ Hydrolysis/ Urine/ Serum/ Plasma/ Blood circulation/ Protein denaturation/ Fats/ Lipids/ Fatty acids/ Glycerides/ Triglycerides/ Dietary fats | Postprandial period  Meals  Beverages |
| Key words | Milk  Milk proteins  Milk fat | Heat* treat*  Heat* process*  UHT  Ultra-high temperature  Pasteuriz*  Pasteuris*  ESL  Extended shelf life  Sterilis*  Steriliz* | Gastric digestion  Kinetics  Gastric absorption  Intestinal absorption  Gastrointestinal absorption  Absorption  Protein digestibility Metabolism  Digestion  Nutrients absorption  Protein breakdown  Hydrolys*  Urin*  Serum  Plasma  Blood circulation  Circulat*  Thermal denaturation  Denaturation  Bioavailability  Bioaccessibility  Lipids  Fatty acids  Triglycerides  Glycerides | Postmeal  Postprandial  Meal  Beverage |

Abbreviations: ESL: extended shelf life; UHT: ultra-high temperature

**Supplemental Table S2** EMBASE (ovid) search strategy

|  | Concept 1 | Concept 2 | Concept 3 | Concept 4 |
| --- | --- | --- | --- | --- |
| Concept | Milk | Heat treatments | Proteins and fats absorption | In the next 24 hours |
| Mesh headings | Milk  Milk protein  Protein intake  Milk fat  Fat intake | High temperature  Heat treatment  Pasteurization  Food preservation  Pasteurized milk  UHT milk | Digestion/ Gastrointestinal Absorption/ Intestinal absorption/ stomach absorption /Absorption/ Lipid absorption/ Protein degradation/ Gastrointestinal Motility/ Protein intake/ Amino acid/ Plasma protein/ Kinetics/ Metabolism/ Bioavailability/ Hydrolysis/ Urine/ Serum/ Plasma/ Circulation/ Protein denaturation/ Denaturation/ Fat/ Lipid/ Fatty acid/ Acylglycerol/ Triacylglycerol/ Dietary fats | Postprandial state  Meal  Beverages |
| Key words | Milk  Milk protein*  Milk fat* | Heat* treat*  Heat* process*  UHT  Ultra-high temperature  Pasteuriz*  Pasteuris*  ESL  Extended shelf life  Sterilis*  Steriliz* | Gastric digestion  Kinetics  Gastric absorption  Intestin* absorption  Gastrointestinal absorption  Absorption  Protein digestibility Metabolism  Digestion  Nutrient* absorption  Protein* absorption  Lipid* absorption  Protein* breakdown  Hydrolys*  Protein* degradation  Urin*  Serum  Plasma  Blood circulation  Circulat*  Thermal denaturation  Denaturation  Bioavailability  Bioaccessibility  Lipid*  Fatty acid*  Triglycerides  Glycerides | Postmeal  Postprandial  Meal  Beverage |

Abbreviations: ESL: extended shelf life; UHT: ultra-high temperature

**Supplemental Table S3** Cochrane Library search strategy

|  | Concept 1 | Concept 2 | Concept 3 | Concept 4 |
| --- | --- | --- | --- | --- |
| Concept | Milk | Heat treatments | Proteins and fats absorption | In the next 24 hours |
| Mesh headings | Milk  Milk protein  Dietary proteins  Dietary fats | Hot temperature  Pasteurization  Food preservation | Digestion/ Gastrointestinal Absorption/ Intestinal absorption/ Gastric absorption /Absorption/ Proteolysis/ Gastrointestinal Motility/ Dietary Proteins/ Amino acids/ Blood Proteins/ Protein Digestion/ Kinetics/ Metabolism/ Biological Availability/ Hydrolysis/ Urine/ Serum/ Plasma/ Blood circulation/ Protein denaturation/ Fats/ Lipids/ Fatty acids/ Glycerides/ Triglycerides/ Dietary fats | Postprandial period  Meals  Beverages |
| Key words | Milk  Milk protein*  Milk fat* | Heat* treat*  Heat* process*  UHT  Ultra-high temperature  Pasteuriz*  Pasteuris*  ESL  Extended shelf life  Sterilis*  Steriliz* | Gastric digestion  Kinetics  Gastric absorption  Intestinal absorption  Gastrointestinal absorption  Absorption  Protein digestibility Metabolism  Digestion  Nutrients absorption  Protein breakdown  Hydrolys*  Urin*  Serum  Plasma  Blood circulation  Circulat*  Thermal denaturation  Denaturation  Bioavailability  Bioaccessibility  Lipid*  Fatty acid*  Triglyceride*  Glyceride* | Postmeal  Postprandial  Meal  Beverage |

Abbreviations: ESL: extended shelf life; UHT: ultra-high temperature

**Supplemental Table S4** Scopus search strategy^1^

|  | Concept 1 | Concept 2 | Concept 3 | Concept 4 |
| --- | --- | --- | --- | --- |
| Concept | Milk | Heat treatments | Proteins and fats absorption | In the next 24 hours |
| Key words | Milk OR “Milk protein*” OR “Milk fat*” | “Heat* treat*” OR “Heat* process*” OR UHT OR “Ultra high temperature” OR Pasteuriz* OR Pasteuris* OR ESL OR “Extended shelf life” OR Sterilis* OR Steriliz* | “Gastric digestion” OR “Kinetics” OR “Gastric absorption” OR “Intestinal absorption” OR “Gastrointestinal absorption” OR Absorption OR “Protein digestibility” OR Metabolism OR “gastrointestinal motility” OR Digestion OR “Nutrients absorption”  OR “Protein breakdown” OR Proteolysis OR Hydrolys* OR Urin* OR Serum OR Plasma OR “Blood circulation”OR Circulat* OR “Thermal denaturation” OR  Denaturation OR Bioavailability OR Bioaccessibility OR Lipid* OR “Fatty acid*” OR Triglycerides  OR Glycerides | Postmeal OR Postprandial OR Meal* OR Beverage* |

Abbreviations: ESL: extended shelf life; UHT: ultra-high temperature

^1^No MESH terms were used during the Scopus search, and only keywords used.
